# Integrated analysis of whole-exome sequencing and transcriptome profiling in males with autism spectrum disorders

**DOI:** 10.1186/s13229-015-0017-0

**Published:** 2015-04-15

**Authors:** Marta Codina-Solà, Benjamín Rodríguez-Santiago, Aïda Homs, Javier Santoyo, Maria Rigau, Gemma Aznar-Laín, Miguel del Campo, Blanca Gener, Elisabeth Gabau, María Pilar Botella, Armand Gutiérrez-Arumí, Guillermo Antiñolo, Luis Alberto Pérez-Jurado, Ivon Cuscó

**Affiliations:** Department of Experimental and Health Sciences, Universitat Pompeu Fabra, C/Doctor Aiguader 88, 422, Barcelona, 08003 Spain; Hospital del Mar Research Institute (IMIM), C/Doctor Aiguader 88, Barcelona, 08003 Spain; Centro de Investigación Biomédica en Red de Enfermedades Raras (CIBER-ER), C/ Monforte de Lemos 3-5, Madrid, 28029 Spain; Quantitative Genomic Medicine Laboratories (qGenomics), C/Doctor Aiguader 88, 422, Barcelona, 08003 Spain; Medical Genome Project, Genomics and Bioinformatics Platform of Andalusia (GBPA), C/Albert Einstein, Cartuja Scientific and Technology Park, INSUR Builiding, Sevilla, 41092 Spain; Pediatric Neurology, Hospital del Mar, Passeig Marítim 25-29, Barcelona, 08003 Spain; Servicio de Genética, Hospital Vall d’Hebron, Passeig Vall d’Hebron, 119-129, Barcelona, 08015 Spain; Genetics Service, BioCruces Health Research Institute, Hospital Universitario Cruces, Plaza de Cruces 12, Barakaldo, Bizkaia 48093 Spain; Pediatrics Service, Corporació Sanitària Parc Taulí, Parc Taulí 1, Sabadell, 08208 Spain; Pediatric Neurology, Hospital de Txagorritxu, C/José de Atxotegui s/n, Victoria-Gasteiz, 01009 Spain; Department of Genetics, Reproduction and Fetal Medicine, Institute of Biomedicine of Seville (IBIS), University Hospital Virgen del Rocío/CSIC/University of Seville, Avda Manuel Siurot s/n, Sevilla, 41013 Spain

**Keywords:** ASD, Whole-exome sequencing, CNV, SNV

## Abstract

**Background:**

Autism spectrum disorders (ASD) are a group of neurodevelopmental disorders with high heritability. Recent findings support a highly heterogeneous and complex genetic etiology including rare *de novo* and inherited mutations or chromosomal rearrangements as well as double or multiple hits.

**Methods:**

We performed whole-exome sequencing (WES) and blood cell transcriptome by RNAseq in a subset of male patients with idiopathic ASD (*n* = 36) in order to identify causative genes, transcriptomic alterations, and susceptibility variants.

**Results:**

We detected likely monogenic causes in seven cases: five *de novo* (*SCN2A*, *MED13L*, *KCNV1*, *CUL3*, and *PTEN*) and two inherited X-linked variants (*MAOA* and *CDKL5*). Transcriptomic analyses allowed the identification of intronic causative mutations missed by the usual filtering of WES and revealed functional consequences of some rare mutations. These included aberrant transcripts (*PTEN*, *POLR3C*), deregulated expression in 1.7% of mutated genes (that is, *SEMA6B*, *MECP2*, *ANK3*, *CREBBP*), allele-specific expression (*FUS*, *MTOR*, *TAF1C*), and non-sense-mediated decay (*RIT1*, *ALG9*). The analysis of rare inherited variants showed enrichment in relevant pathways such as the PI3K-Akt signaling and the axon guidance.

**Conclusions:**

Integrative analysis of WES and blood RNAseq data has proven to be an efficient strategy to identify likely monogenic forms of ASD (19% in our cohort), as well as additional rare inherited mutations that can contribute to ASD risk in a multifactorial manner. Blood transcriptomic data, besides validating 88% of expressed variants, allowed the identification of missed intronic mutations and revealed functional correlations of genetic variants, including changes in splicing, expression levels, and allelic expression.

**Electronic supplementary material:**

The online version of this article (doi:10.1186/s13229-015-0017-0) contains supplementary material, which is available to authorized users.

## Background

Autism spectrum disorders (ASD) [OMIM 209850] are defined as a group of neurobehavioral syndromes characterized by deficits in social interaction, impaired communication skills and restricted, stereotypical and ritualized patterns of interests and behavior, typically appearing before the age of 3. Throughout the last decades, the prevalence of ASD has risen from the historically estimated proportion of 4/10,000 to approximately 1/110 children (2008) [1-3] with a ratio four times higher in males than females [4]. It is still debated how much of this increase is related to diagnostic improvements, raised awareness towards ASD, or emerging environmental factors [5,6]. ASD are among the most heritable neuropsychiatric disorders, given that concordance rates in monozygotic twins are 90% and siblings have an approximately 50-fold increased risk of ASD. ASD are found in association with comorbid genetic conditions in 10% of cases and are considered complex multifactorial disorders involving multiple genes [7,8]. Currently, the etiology can be established in only 30% of the cases and remains unknown for most patients.

The technological improvements of the last decade have lead to tremendous advances in understanding the genetic basis of ASD, revealing a high degree of genetic heterogeneity. Clinical application of molecular karyotyping has shown that 5% to 10% of patients carry chromosomal rearrangements and that the burden of rare and *de novo* smaller copy number variants (CNVs) is higher among ASD patients than controls. However, since many of these variants show incomplete penetrance and variable phenotypic expression, the best model to explain most ASD cases would be oligogenic with a probable environmental contribution. Until now, most genomic studies on ASD using next-generation sequencing (NGS) have focused on coding regions and have analyzed trios in an effort to identify *de novo* mutations [9-14]. Only a few studies investigated rare inherited variation [15-18]. The reported data suggest a contribution of *de novo* disruptive mutations in the genetic etiology of ASD, with hundreds of genes implicated and only a few of them recurrently mutated in unrelated cases (*CHD8* [*MIM 610528*], *DYRK1A* [*MIM 600855*], *GRIN2B* [*MIM 138252*], *KATNAL2* [*MIM 614697]*, *POGZ* [MIM 614787], and *SCN2A* [*MIM 182390*])*.* Besides *de novo* disruptive mutations, comparison of rates of rare variation across the whole genome in cases *versus* controls have yielded no significant associations. These findings support previous hypothesis which suggested that a large number of genes confer risk to ASD and reinforce the idea that much larger cohorts will be necessary to carry out this type of analyses [19]. The identification of new genes involved in ASD will eventually lead to the definition of common effects of genetic variants and possibly ASD biomarkers and biological signatures. Biology system tools such as interaction networks are important to detect common deregulated pathways and expression networks implicated in the disease.

An additional approach to identify genetic variants associated with a phenotype and to understand the biological effects resulting from rare genetic variation could be derived from observing the transcriptomic consequences of genetic variation [20]. To this end, we have analyzed 36 Spanish male patients with idiopathic ASD by whole-exome sequencing (WES) to define causative or susceptibility variants for ASD and their transcriptomic consequences by RNAseq. In addition to the identification of likely monogenic cases, we also studied the accumulation of rare genetic variation which could result in putatively common functional consequences.

## Methods

### Sample selection

We studied 36 unrelated males with a diagnosis of idiopathic ASD selected from a Spanish cohort of 324 patients. All cases except two were sporadic. All patients had a confirmed diagnosis of one of the categories of ASD listed in the Diagnosis and Statistical Manual of Mental Diseases IV (DSM-IV), categorized according to the Spanish version of ADI-R (Autism Diagnostic Interview-Revised), and the Wechsler Intelligence Scale for Children or Wechsler Adult Intelligence Scale. All patients had an extensive clinical and molecular evaluation including fragile X testing and molecular karyotype (either BAC, oligo, or SNP array) with normal results. The study was approved by the Clinical Research Ethics Committee of the centers involved (CEIC-Parc Salut Mar), and informed consent for participation was obtained from the parents or legal caregivers. Blood samples were obtained, and genomic DNA was extracted by the salting out method using the Puregene® DNA Purification Kit (Gentra Systems, Big Lake, MN, USA). Parental and familial samples were obtained from the available relatives who gave informed consent.

### Whole-exome capture and sequencing

The exome portion of the genome was enriched using NimbleGen EZ Exome V2.0 capture kit (Roche Applied Science, Madison, WI, USA). Gene and exon annotations for SeqCap EZ Human Exome Library came from RefSeq (Jan 2010), CCDS (Sept 2009), and miRBase (v.14, September 2009). A total of approximately 30,000 coding genes (approximately 300,000 exons, total size 36.5 Mb) were targeted by the design, and a total of 44.1 Mb were covered by the probes. Final libraries were then sequenced on an ABI Solid 4 platform (Life Technologies, Carlsbad, CA, USA). Single-end sequences were obtained with a read length of 50 bp.

### Variant calling, annotation, and prioritization

A pipeline for data alignment using BFAST [21] and GATK [22] algorithms was applied to the sequencing data following standard parameters. Briefly, sequences were aligned to the latest version of the human genome (hg19), PCR duplicates were marked and removed, and quality scores of alignments were recalibrated. Single nucleotide variants (SNV) and indel calls were only considered if positions had a depth of coverage of at least 10×, and heterozygous positions were only called when a minimum of 20% of the reads showed the variant (AB between 0.2 and 0.8). In order to minimize technical artifacts, we removed variants that appeared in more than two samples, even if they were present in a single read or had an AB ratio lower than 0.2. Annotation of variants was performed using ANNOVAR (http://www.openbioinformatics.org/annovar/), taking into account the variant frequency in control databases: dbSNP135 (http://www.ncbi.nlm.nih.gov/SNP/), Exome Variant Server (EVS) (http://evs.gs.washington.edu/EVS/), and an in-house database of 90 Spanish controls. The nature of the changes was assessed by PolyPhen and Condel (http://bg.upf.edu/fannsdb/) protein effect prediction algorithms [23]. To distinguish the putative disease-causing variants, we established the following criteria: (1) we selected only non-synonymous variants; (2) under a dominant model, we excluded variants previously described in the general population (dbSNP135, EVS, 1000 Genomes (http://browser.1000genomes.org) and Spanish controls); (3) under a recessive model, we removed variants with a minor allele frequency (MAF) >0.002 and only considered genes with homozygous or compound heterozygous mutations; (4) we discarded variants present in loss of function tolerant genes as previously described [24]; and (5) we manually inspected recurrent variants and indel calls to exclude false positives using Integrative Genomics Viewer (IGV) [25].

We used the XHMM algorithm to call CNVs, based on measurement of the read depth per target region (GATK). We followed the standard steps as described in the online tutorial. We applied the same filters previously described [26]: XHMM quality score (SQ) ≥65, exons spanned ≥3, and estimated CNV length ≥1kB. We focused our analysis on rare CNVs, so we excluded CNVs overlapping with polymorphic variants reported in Database of Genomic Variants (DGV) (http://dgv.tcag.ca/dgv/app/home).

### Validation

We used Sequenom genotyping (iPLEX Gold platform, San Diego, CA, USA) and Sanger sequencing by capillary electrophoresis (ABI PRISM 7900HT, Applied Biosystems, Foster City, CA, USA) to perform validation and segregation studies. To genotype the selected variants, we designed primers (PRIMER 3 application) (http://www.bioinformatics.nl/cgi-bin/primer3plus/primer3plus.cgi/) and used standard PCR conditions. For CNV validation, we used multiple ligation probe amplification (MLPA) with custom probes in the target region. MLPA reactions were carried out under standard conditions. The relative peak height method was used to determine the copy number status. We analyzed samples from the proband and both parents as well as from other relatives when available.

### Paternity testing

We performed microsatellite genotyping of trios to corroborate paternity on patients with *de novo* mutations. We selected highly heterozygous microsatellites markers randomly distributed in different autosomal chromosomes. PCR products were amplified under standard conditions, and fragments were separated and analyzed by high-resolution electrophoresis using GeneMapper software (ABI 3100, Applied Biosystems, Foster City, CA, USA).

### X-chromosome inactivation analysis

To determine the X-chromosome inactivation pattern (XCI), we examined the differential methylation state of nearby *HpaII* sites of the polymorphic CAG repeat in exon 1 of the human androgen receptor gene (*AR* [MIM 313700]) located at Xq13. Following digestion with methylation sensitive restriction endonuclease *HpaII*, the region was amplified by PCR with a FAM labeled forward primer [27,28]. The digested and not digested PCR products were analyzed in an ABI PRISM 3100 Genetic Analyzer. For quantitative analysis, trace data were retrieved using the accompanying software (GeneMapper, Applied Biosystems, Foster City, CA, USA). The degree of XCI skewing was calculated as the fractional peak height ratio (expressed as %) for the more strongly amplified allele. XCI was considered significantly skewed if the ratio exceeded 90:10.

### Transcriptome sequencing

Peripheral mononuclear cells (PBMCs) from whole blood of 36 studied ASD patients were isolated using a ficoll density gradient (Lymphoprep™, STEMCELL Technologies, Vancouver, British Columbia, Canada). Total RNA was extracted using Trizol (Life Technologies, Carlsbad, CA, USA) following a standard protocol. The quality and yield of the isolated RNA was assessed using a NanoDrop8000 Spectrophotometer (Thermo Fisher Scientific, Waltham, MA, USA) and Agilent 2100 Bioanalyzer (Agilent Technologies, Santa Clara, CA, USA). Transcriptome sequencing was performed on a HiSeq 2000. Paired-end sequences were obtained at a read length of 100 bp with 57,792,576 mean read pairs per sample. Sequences were aligned to the NCBI build 37 human genome reference using TopHat [29] and Bowtie [30] to map the inter-exon splice junctions. Cufflinks [31] and htseqcount [32] were used to estimate the expression of the transcripts (FPKM - fragment per kilobase of transcript per million fragments mapped- and read counts). We used the ComBat algorithm (http://www.bu.edu/jlab/wp-assets/ComBat/Abstract.html) (package sva R) to remove batch effect and to obtain the z-score of expressed genes.

### Allele-specific expression analysis

In order to study extreme imbalances of allelic expression, either allele-specific or preferential expression, we selected heterozygous SNPs (dbSNP135) identified by WES in each patient with a minimum depth of coverage of 15 and an AB ratio between 0.3 and 0.7. SNPs in known segmental duplications or pseudogenes according to UCSC hg19 (http://genome.ucsc.edu/cgi-bin/hgGateway) ‘Segmental Dups’ and ‘Retroposed Genes’ tracks were excluded from the analyses. We then extracted the number of RNAseq reads mapped to each position and selected only highly covered positions (at least 20×). We classified each SNP expression according to its AB ratio, being biallelic when the AB ratio was between 0.1 and 0.9 and monoallelic when predominantly the reference or alternative allele were expressed (AB > 0.9 or <0.1). When all SNPs of a gene were monoallelic, we classified the gene as having monoallelic expression, whereas genes with biallelic SNPs were considered to have biallelic expression.

### Isoform-splicing analysis

The aligned reads were processed by Cufflinks, using a supplied reference annotation (Homo_sapiens.GRCh37.68.gtf) to guide RABT assembly. Assembled transcripts were then analyzed by Cuffcompare to compare isoforms across all samples. We then selected novel isoforms (defined by Cufflinks by class code j) with an expression >2 FPKM and matched them to rare variants found by exome sequencing in the same patient.

### RNA editing analysis

We first applied stringent filtering criteria to remove RNAseq false positive calls (DP > 10, SB ≤ 0.1, HRun < 8, ReadposRankSum ≥ 2.0, BaseQRankSum ≥ 2) and then annotated depth of coverage of the same positions according to exome sequencing. We selected only positions with a depth of coverage of at least 15× and that were not called by exome sequencing, excluding all variants described in control databases (dbSNP135, Exome Variant Server) and those present in another sample. We manually revised the remaining variants to discard false positive calls.

### Pathway enrichment analysis

To identify common deregulated mechanisms affected by rare genetic variants, we performed pathway enrichment analyses using the publicly available ConsensusPathDB database (CPDB) (http://cpdb.molgen.mpg.de/). CPDB incorporates interaction data from different categories including metabolic and signaling reactions, physical protein and genetic interactions, or gene regulatory interactions. Statistical analyses were performed using the CPDB overrepresentation analysis option, with four categories of predefined genes (network neighborhood-based, pathways-based, Gene Ontology-based and protein complex-based gene sets). For each of the predefined sets, a *P*-value was calculated according to the hypergeometric test based on the number of physical entities present in both the predefined set and user-specified list of physical entities. For pathway-based sets, we used the default *P*-value threshold of 0.01. We used the default gene background defined by CPDB as the number of entities that are annotated within the category of the provided gene. We then compared overrepresented pathways among rare WES variants in ASD samples with respect to 55 Spanish non-ASD samples.

## Results

**Figure 1:**
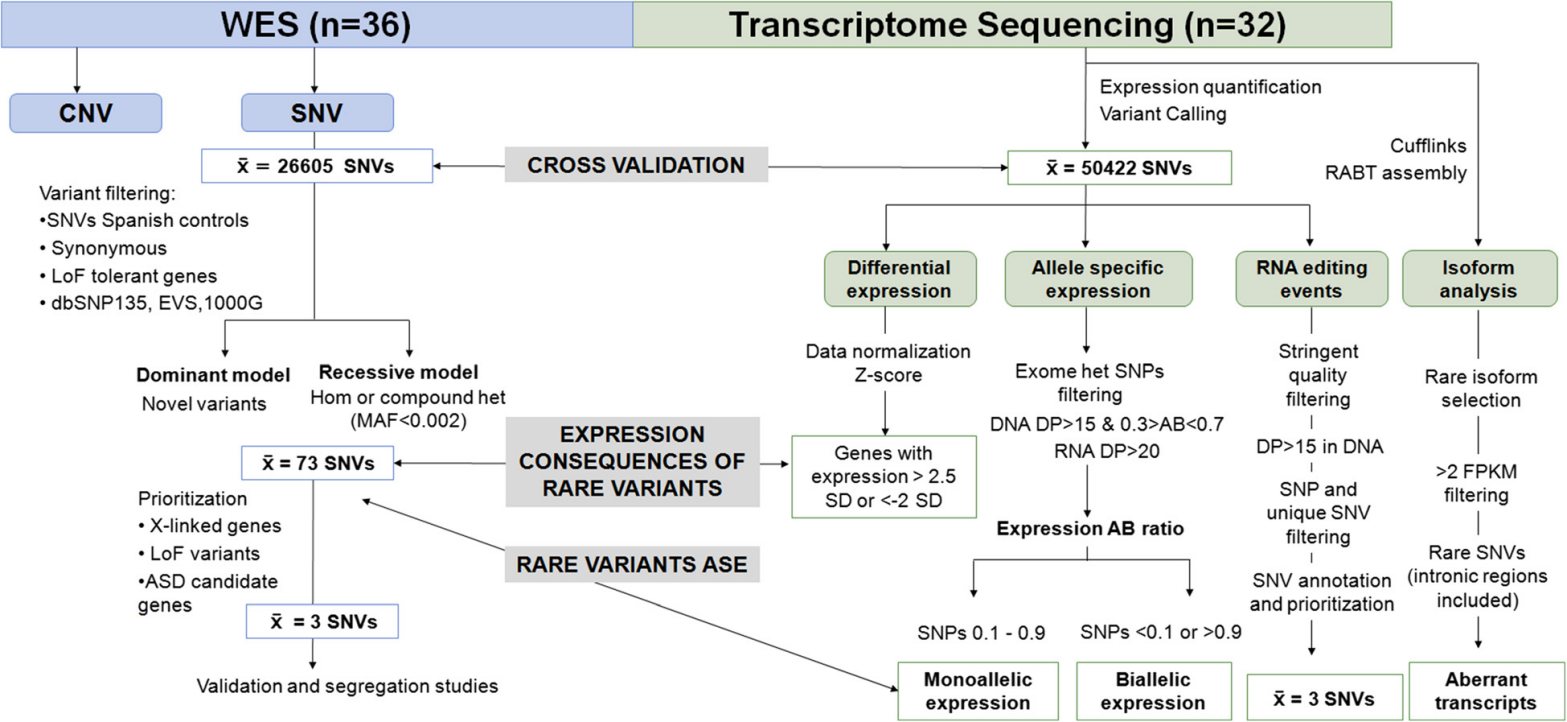
**Workflow of the Omics approach used to define the likely pathogenic genetic variants and transcriptomic consequences in the studied ASD cohort.**

In order to perform the integrative analysis of next-generation sequencing data, we followed the workflow shown in Figure 1.

### WES general overview

Overall, a mean depth of coverage of 40× was obtained and an average of 26,605 variants per sample was observed. After filtering, considering only non-synonymous, frameshift, or splice site rare variants (MAF dominant = 0; recessive <0.002), we selected a total of 2,626 calls, with an average of 73 rare events per patient (Figure 1). We classified the variants into missense and loss of function variants (LoF), a category which included splicing, non-sense, and frameshift mutations. Most rare variants were missense (91%), while six per sample on average (9%) were LoF (Additional file 1: Figure S1). The 2,626 rare variants were distributed in 2,205 genes, most of them carrying just one mutation, although some accumulated a high number of rare variants, with extreme cases such as *MUC16* (MIM 606154) and *TTN* (MIM 188840) with 8 and 17 different mutations, respectively.

We considered likely pathogenic mutations all LoF variants, predicted damaging variants on the X chromosome or in autosomal candidate genes (according to the two main ASD databases, SFARI and AUTkB, see Additional file 2: Table S1) [33,34] and variants which involved a detectable change in the gene expression. To discriminate putative ASD pathogenic genes from non-related loci which accumulate LoF without any pathological consequence [24], we analyzed the distribution of this type of variants in the population with European ancestry described in the EVS (*n* ~ 4,300), which contains exome sequencing data from individuals with unrelated disorders. We only considered as candidates the LoF variants that were found in genes with no LoF mutations in the general population. We finally selected 121 variants, with an average of 3 per patient. All novel variants absent in dbSNP137 (*n* = 109) were validated by Sequenom and/or Sanger sequencing, and segregation studies were performed using parental and other relatives’ samples when available (Additional file 2: Table S2).

### Autosomal variants

We studied the inheritance of 103 likely damaging variants (70 LoF and 33 missense) in candidate autosomal genes using parental samples. Only 5/103 rare variants (4.8%) were *de novo*, and the remaining (95.2%) were inherited from unaffected parents. *De novo* LoF mutations were detected in *SCN2A* (p.R583X), *MED13L* [*MIM 608771*] (c.1708_1709delAG), *KCNV1* [*MIM 608164*] (c.1391_1392delAT), and *ADIPOR2* [MIM 607946] (p.R31X) in four different patients. Three of *de novo* LoF mutations are probably pathogenic, since they are predicted to result in truncated proteins. However, the *de novo* variant in *ADIPOR2* found in a familial case (ASD_16) was not present in the affected sibling, excluding it as main ASD cause in this family although it could contribute as a modifier of the phenotype [35]. Additionally, we detected a *de novo* missense mutation affecting a highly conserved residue in *CUL3* [*MIM 603136*] (*p.H719R*). Paternity was confirmed in all cases. Notably, none of these genes present a LoF variant in the general population. Detailed information about the phenotype of these patients is shown in Table 1.

**Table 1:** **Summary of the phenotypic features of ASD patients and relevant findings of the study**

**Patient**	**Birth year**	**Intellectual disability**	**ADIR classification**	**Seizure episode**	**Dysmorphic features**	**Functional language**	**Other medically relevant conditions**	**Molecular diagnosis**	**Specific ASD overrepresented pathways**
ASD_1	1992	Severe	AUT	No	No	No	Obesity	*CDKL5* (p. P647L)	
ASD_2	1995	Severe	PDD-NOS	No	No	No	Hypermetropia		Agrin in postsynaptic differentiation
									Elastic fiber formation
									Erk and pi-3 kinase for collagen binding in corneal epithelia
									Integrin cell surface interactions
									Molecules associated with elastic fibers
									Ucalpain and friends in cell spread
ASD_3	1993	Mild	AUT	Yes	No	No			
ASD_4	1995	Mild	AUT	Yes	No	No			
ASD_5	1997	Moderate	AUT	No	No	Yes	ADHD	*SCN2A* (p.R583X)	
ASD_6	1998	Mild	AUT	No	No	Yes			
ASD_7	1999	Mild	BS	No	Yes	Yes	Sleep disturbance, ADHD	*CUL3* (p.H719R)	
ASD_8	1991	None	AUT	No	No	Yes			Integrin cell surface interactions
ASD_9	2000	Moderate	AUT	No	No	No			
ASD_10	2000	Severe	AUT	No	No	No			
ASD_11	1999	Severe	AUT	No	Yes	No	Sleep disturbance, hypotonia, umbilical hernia	*MED13L* (*c.1708_1709delAG*)	
ASD_12	2002	Moderate	AUT	No	Yes	No	ADHD		
ASD_13	2001	None	AUT	No	No	Yes			Elastic fiber formation
									Signaling events mediated by HDAC class II
									Sumoylation by RanBP2 in transcriptional repression
ASD_14	2000	Severe	AUT	No	No	No	ADHD	*KCNV1* (c.1391_1392delAT)	
ASD_15	2001	Mild	AUT	Yes	Yes	Yes			
ASD_16	2001	Moderate	AUT/PDD-NOS	Yes	No	Yes	Mild psychosis, aggressive behavior	*MAOA* (c.1438-2A > G)	
ASD_17	2000		AUT	No		Yes			Beta1 integrin cell surface interactions
									Beta3 integrin cell surface interactions
									Integrin cell surface interactions
									Integrins in angiogenesis
									Signaling by PDGF
ASD_18	2003		BS	No		No			Axon guidance
									NCAM signaling for neurite out-growth
									Ucalpain and friends in cell spread
ASD_19	2001		AUT	No		No			Beta3 integrin cell surface interactions
									Integrin cell surface interactions
									Signaling by PDGF
ASD_20	1998		AUT	No		No			Elastic fiber formation
									Huntington’s disease
									Molecules associated with elastic fibers
ASD_21	1997	Severe	AUT	No	Yes	No	Sleep disturbance		
ASD_22	2000	Moderate	AUT	No	No	No			
ASD_23	2005	None	AUT	No	No	NO			
ASD_24	2000	Severe	AUT	Yes	No	No	ADHD		
ASD_25	2002	Mild	AUT	No	Yes	Yes			
ASD_26	2000	Mild	AUT	Yes	No	No			PI3K-Akt signaling pathway
ASD_27	2005		AUT	No		No	Trimethylaminuria		Beta1 integrin cell surface interactions
									Erk and pi-3 kinase for collagen binding in corneal epithelia Integrins in angiogenesis
									PI3K-Akt signaling pathway
									Scavenging by class A receptors
									Striated muscle contraction
ASD_28	1995		AUT	Yes		No	Moderate obesity, myopia		Scavenging by class A receptors
									Signaling events mediated by HDAC Class II
									Sumoylation by RanBP2 in transcriptional repression Superpathway of cholesterol biosynthesis
ASD_29	1999	Severe	AUT	No	No	No			Huntington’s disease
ASD_30	1997	Severe	AUT	Yes	No	No			Termination of O-glycan biosynthesis
ASD_31	2008	None	AUT	No	No	Yes			Striated muscle contraction
ASD_32	2004	Mild	AUT	No	No	Yes			
ASD_33	1990		AUT	No		No			Agrin in postsynaptic differentiation
									L1CAM interactions
									Ucalpain and friends in cell spread
ASD_34	2001		AUT	No		Yes			Superpathway of cholesterol biosynthesis
									Termination of O-glycan biosynthesis
ASD_35	2003		PDD-NOS	No		Yes			Axon guidance
									Integrin cell surface interactions
									L1CAM interactions
									NCAM signaling for neurite out-growth
ASD_36	1999	Severe	AUT	No	No	No	Macrocephaly	*PTEN* (*c.239-21G > C*)	

*De novo* non-sense *SCN2A* mutations have been already described in several cases of ASD [10,36-38]. The mutation we report is found in patient ASD_5; it results in a stop codon located after the first transmembrane domain, leading to a truncated protein with only one of the four transmembrane domains. The two base-pair frameshift deletion in *MED13L* in patient ASD_11 generates a premature stop codon and a truncated protein lacking its main part. The frameshift mutation in *KCNV1* in patient ASD_14 produces a stop codon shortly after, resulting in a prematurely truncated protein and abolishing the last cytoplasmatic domain. *CUL3*, mutated in ASD_7, has already been reported as ASD candidate, given that two *de novo* non-sense mutations were identified in previous work, a result that is highly unlikely by chance [11,39].

All remaining variants were inherited from unaffected parents, suggesting either incomplete penetrance of these mutations or a contribution as an additive effect in those patients (Additional file 2: Table S2).

### X-linked variants

We validated and performed segregation studies on all X-linked variants that had not been previously reported or were found in candidate genes. We extended the study to the available healthy male family members to discard variants that did not segregate with the phenotype (Additional file 2: Table S3).

We found two X-linked mutations in *MAOA* (MIM 309850) and *CDKL5* (MIM 300203). A splicing mutation (c.1438-2A > G) in *MAOA* was detected in the autistic proband (ASD_16) and later also in his brother diagnosed with pervasive developmental disorder not otherwise specified (PDD-NOS) (Table 1). The mother (mutation carrier) and maternal grandmother presented with a depressive and bipolar disorder, respectively. Unfortunately, no DNA was available from the grandmother to analyze the presence of the variant. The mutation led to aberrant splicing through the use of a cryptic splice site within intron 15 of *MAOA* that was confirmed by RT-PCR of blood mRNA from the patients (Additional file 1: Figure S2). We also determined the levels of catecholamines and related metabolites in urine of these patients and their mother. These studies showed altered levels of several neurotransmitters in both affected brothers [increased serotonine (2×), normetanephrine (2.4×), and 5-HIAA (0.45×)] and very mild in their mother (Additional file 2: Table S4), confirming the alteration of the monoamine metabolism.

A missense variant affecting a highly conserved residue (p.P647L) in the *CDKL5* gene was also found in patient ASD_1. Familial studies showed that the mutation was inherited from the mother and also present in the healthy sister but not in two healthy uncles. *CDKL5* is a member of the Ser/Thr protein kinase family and encodes a phosphorylated protein with protein kinase activity. Mutations in this gene have been associated with X-linked infantile spasm (ISSX) and atypical Rett syndrome (RTT) [40-43].

We also carried out X chromosome inactivation (XCI) analyses on blood cell DNA from all 36 mothers of the probands, as an additional tool to predict putative pathogenicity of X-linked mutations. Six mothers were non-informative, 25 had a random XCI, and 5 showed a skewed XCI with ratios >90%. Among the XCI skewed mothers, only two presented rare variants in the X chromosome, being one also detected in a healthy uncle and the other inactivated preferentially the non-transmitted allele. In the *CDKL5* mutation carriers, the XCI study was not informative in the mother but the proband’s sister showed a selective inactivation of the maternally inherited mutated allele.

### Recurrently mutated genes in ASD

We studied in detail the presence of recurrently mutated genes and the functional consequences resulting from the accumulation of rare genetic variants to identify common mechanisms involved in ASD etiology.

Since most putative pathogenic variants detected were inherited, we focused on genes mutated in more than one case of our ASD cohort. We found a total of 297 recurrently mutated genes, although most of them also showed a significant load of rare variants in the general population (EVS data). A total of 15 genes lacking LoF mutations in publicly available data from EVS were mutated with at least one LoF in more than one ASD case of our cohort (Additional file 2: Table S5). The list includes two genes with *de novo* variants in a patient and a missense mutation in the second patient (*SCN2A* and *MED13L*). These variants cannot be fully penetrant mutations since they were inherited from healthy progenitors, but might behave as susceptibility factors in multifactorial or multiple-hit models. A proper association study with much larger sample size would be required to draw more clear conclusions about the pathogenicity of the recurrently mutated genes observed in this series.

### Common functional pathways affected by inherited rare mutations in ASD

Previous studies suggest that, in addition to the relatively rare *de novo* and Mendelian forms, the etiology of ASD is mostly multifactorial with multiple genetic hits, mainly rare genetic variants in a few of hundreds of candidate genes [10,11,44]. To further define the potential pathogenic contribution to ASD of the inherited variants and identify common functional consequences, we conducted pathway enrichment analyses for each individual of our cohort excluding those with presumably monogenic forms (*ASD_1*, *ASD_5*, *ASD_7*, *ASD_11*, *ASD_14*, *ASD_16*, and *ASD_36*) (*n* = 29) compared with 55 Spanish non-ASD controls. Using CPDB [45], we found 29 pathways overrepresented in ASD, 21 of them ASD-exclusive and absent in controls (Table 1). Interestingly, some of these pathways have also been previously related with the ASD phenotype, such as the PI3K/Akt signaling and the axon guidance [45-48]. We checked if the genes involved in monogenic cases are also involved in overrepresented pathways. The results show that three of the *de novo* mutated genes are part of overrepresented pathways. *SCN2A* is involved in Axon Guidance and L1CAM interaction pathways and *PTEN* is part of the PI3K-AKt and PDGF signaling pathways. These data underscore the role of those pathways in the pathogenesis of the ASD.

### Rare CNVs in ASD samples

Although all patients had a previous molecular karyotype (either BAC, oligo, or SNP array), we analyzed copy number variants from exome data to identify small rare rearrangements that could have been previously missed. Using the XHMM algorithm and appropriate filtering, we detected a total of 11 rare CNVs (3 deletions and 8 duplications), ranging from 8.2 to 254 Kb. All were validated by MLPA and segregation studies showed that all were inherited from an unaffected progenitor (Additional file 2: Table S6). One of these inherited duplications (patient ASD_9) at chr14q24.2 includes three genes (*DCAF4*, *RBM25* [MIM 612427], *ZFYVE1* [MIM 605471]) and partially disrupts two genes (*PSEN1* [MIM 104311] and *DPF3* [MIM 601672]). *PSEN1* encodes a protein that regulates the processing of amyloid precursor protein (APP) and is mutated in familial forms of Alzheimer’s disease while *DPF3* function is associated with the BAF chromatin remodeling complex to promote transcription during development [49]. In addition, we detected two unrelated ASD individuals carrying a duplication of the X-Y pseudoautosomal *ASMT* [MIM 300015, 402500] previously associated with autism phenotype and sleep disturbance [50-52].

### Transcriptomic alterations in blood mRNA of ASD

Good-quality blood transcriptome by RNAseq was available from 32 patients. Up to 64% of protein coding genes (14,114 of 22,057, according to Ensembl) were expressed at >0.3 FPKM, which is considered a robust threshold of gene expression [53]. No relevant differential transcriptomic pattern was evident in any group of samples by principal component analysis. We also compared individual samples against the rest in order to search for individual patterns of aberrant expression. The detectable variable genes per patient were unrelated and did not result in a common biological signature.

The integrative study of WES and RNAseq was limited to the almost 1/3 (30%) of the positions identified as rare variants in exome data that had a depth of coverage >10× in RNAseq, the established threshold for comparison. Out of those variants, 88% were also concordantly detected by RNAseq, showing high correlation between the two techniques and demonstrating the calling robustness.

### mRNA expression consequences of rare variation

**Figure 2:**
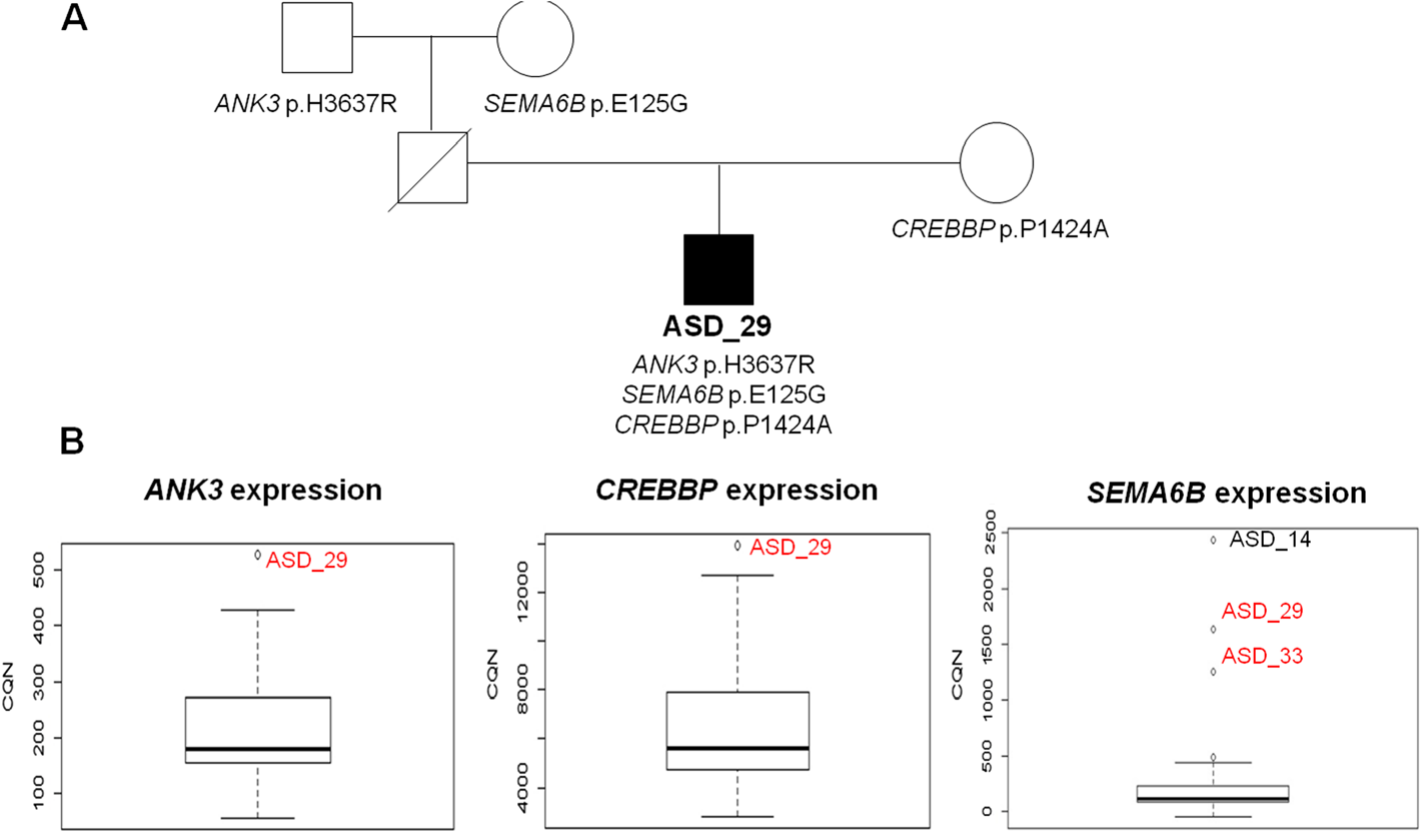
**Quantitative changes in expression associated with rare variants. (A)** Familial pedigree showing segregation of rare mutations. **(B)** Boxplot showing the distribution of expression values and expression outliers corresponding to the patients with rare mutations (in red).

A total of 30 rare variants (1.7% of all expressed) correlated with significant quantitative alterations in expression values of the same gene. All these variants were inherited from healthy progenitors (Additional file 2: Table S7). Among them, 6 were associated with decreased expression (z-score values ≤2), whereas 24 were associated with overexpression (z-score values >2.5). Two rare mutations in *SEMA6B* [MIM 608873] correlated with *SEMA6B* overexpression in two different samples (ASD_29 and ASD_33, Figure 2). Interestingly, ASD_29 carried two additional inherited candidate missense mutations in *ANK3* [MIM 600465] and *CREBBP* [*MIM 600140*], which also correlated with overexpression of the mutated genes (Figure 2). Whereas the *CREBBP* variant was maternally inherited, the other two were inherited from the father, who committed suicide. We also detected a missense mutation (c.C1198T; p.P400S) previously described as a rare SNP in the *MECP2* [*MIM 300005*] that correlated with higher expression of the gene (z-score = 2.16) (Additional file 2: Table S7).

We also evaluated the expression changes of the 22 genes contained in the 11 rare CNVs detected in ASD patients. Only eight genes were expressed in blood at >0.3 FPKM, but we did not detect any significant outlier in expression (z-score values ≤2 or >2.5). However, a global analysis comparing z-score values between genes contained in all detectable deletions and duplications (a total of 26 expressed genes contained in any CNV), showed a tendency for higher expression values for duplications (mean z-score = 0.43) compared to deletions (mean z-score = −0.39) (Additional file 1: Figure S3). Additionally, we detected 10 inherited rare LoF variants resulting in a premature stop codon associated with monoallelic expression of the wild-type allele, suggesting non-sense mediated mRNA decay (Additional file 2: Table S8).

We also searched for possible mRNA editing events in transcriptome data. After strict filtering process and manual curation of the variants in IGV, we did not detect any alteration indicative of mRNA editing. All mismatches between WES and RNAseq were either false negative WES calls or RNA sequencing errors located in the last position of the read or in a homopolymer region.

### Isoform-splicing analysis

RNAseq technology offers the opportunity to identify not only quantitative but also qualitative changes, including novel isoform identification and accurate detection of biased allele expression, in comparison to previous technologies such as expression microarrays.

**Figure 3:**
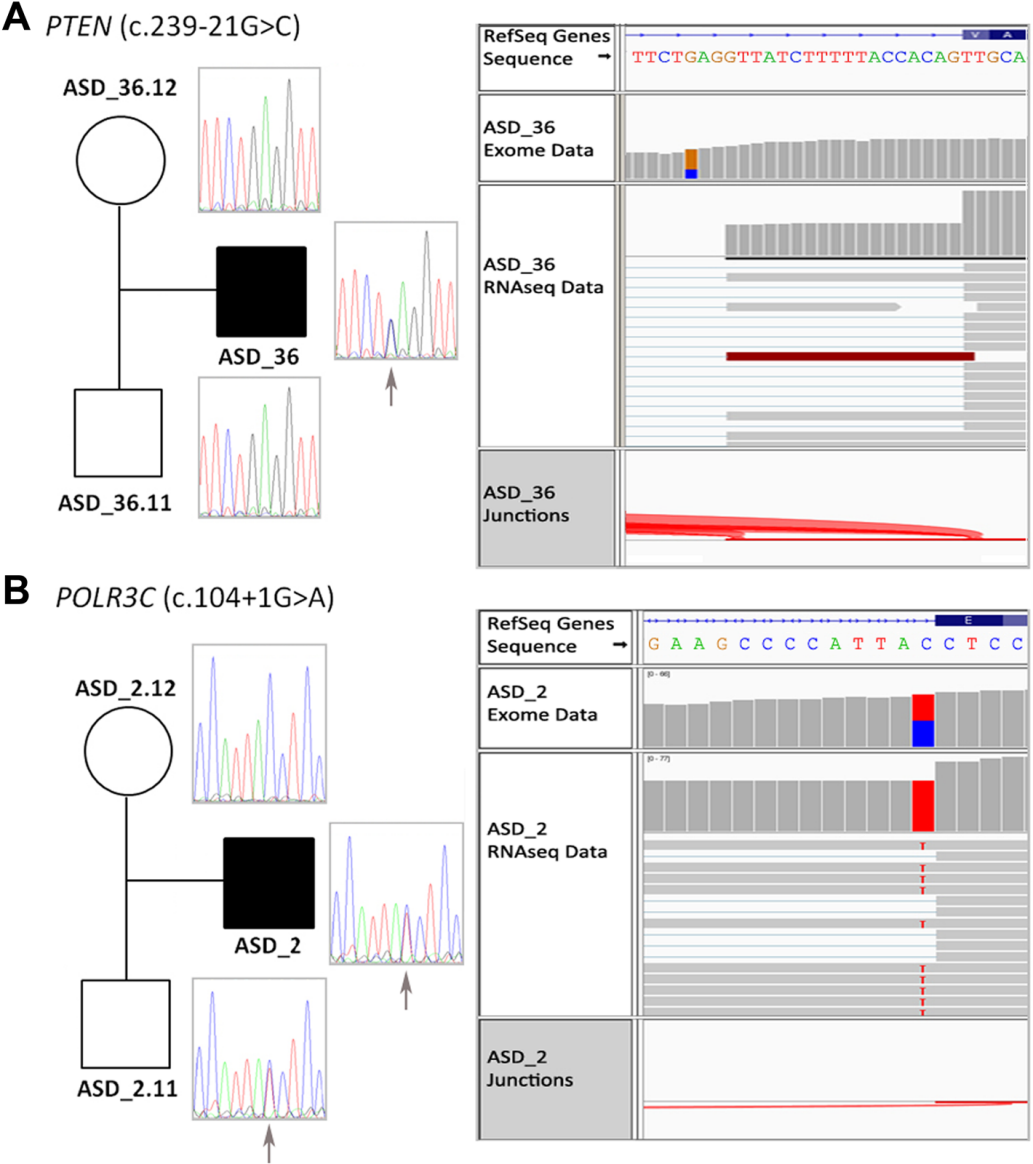
**Segregation analysis by Sanger sequencing and Integrative Genomics Viewer (IGV) pileups in two trios.** **(A)** *de novo* mutation (c.239-21G > C) found in the *PTEN* gene by exome sequencing and the aberrant transcript detected by transcriptome sequencing. **(B)** Paternal inherited mutation that disrupts a splicing donor site in *POLR3C* gene (c.104 + 1G > A) causing retention of an intron.

Isoform analysis by Cufflinks showed two aberrant transcripts due to rare genetic variants. The first was found in the *PTEN* and led to the insertion of an intronic fragment and the incorporation of six new amino acids to the protein. The new exon junction was found in close proximity to a deep intronic variant (c.239-21G > C) that generates a cryptic splice site. Segregation studies showed that it was *de novo* (Figure 3) and only present in patient ASD_36. The second aberrant transcript was found in *POLR3C* and retains an intron that generates a premature stop codon (ASD_2). The aberrant transcript is caused by a mutation that disrupts a splicing donor site (c.104 + 1G > A) inherited from the healthy father.

### Allele-specific expression

To search for extreme imbalances of allelic expression or allele-specific expression, we studied 4,733 well-covered genes with at least one heterozygous SNP not located in a known segmental duplication or a pseudogene. We found 4,426 genes (93%) with biallelic and 81 (2%) with monoallelic expression in all informative samples, while the expression was not homogenous among samples for the remaining 226 genes (5%). Only three genes with consistent monoallelic expression in all samples (*SNRPN* [MIM 182279], *ZDBF2*, and *ZNF597* [MIM 614685]) are known to be imprinted. On the other hand, we identified seven known imprinted genes with biallelic expression in blood, two of them previously reported (*NAA60* [MIM 614246] and *ZFAT* [MIM 610931]) [54-56]. Regarding individual differences, we detected 78 genes with monoallelic expression in a single patient but biallelic in the remaining (at least five informative samples) excluding genes with biased expression (AB ratio >0.8 or <0.2) in at least one sample (Additional file 1: Figure S4). We did not found any individual allelic imbalance in the opposite direction suggestive of loss of imprinting (Additional file 2: Table S9). Nine of the identified genes were previously described to have monoallelic expression in control samples [57-59]. The allelic expression imbalance was found in 26 of 32 samples, affecting between one and eight different genes with no regional clusters. None of these allelic expression imbalances were apparently related to *cis*-effects of the rare mutations or CNVs identified by WES. Interestingly, some of the altered genes have been associated to disease, such as *MTOR* [MIM 601231], *FUS* [MIM 137070], and *TAF1C* [MIM 604905].

## Discussion

Autism spectrum disorders are a group of heterogeneous disorders with a strong genetic component but a complex genetic architecture. This complexity makes genetic diagnosis challenging, with a current diagnostic yield ranging from 15% to 30% [60,61]. Unbiased genome-wide molecular tools such as NGS, with a steadily lowering cost, have a proven efficacy, although they produce genetic and genomic information that cannot be properly interpreted yet. Here, we used WES and blood transcriptome by RNAseq in a selected group of males with idiopathic ASD to detect putative causal genetic variants of this complex disease. Segregation and recurrence analyses along with expression studies were used to better discriminate putatively pathogenic variants from innocuous rare variation.

WES identified several cases with likely monogenic forms of ASD, including four patients with *de novo* variants in strong candidate autosomal genes (11%) and two patients with inherited X-linked mutations (5.6%). Since our study did not include parental exome sequencing, our detection rate of monogenic cases may be underestimated. Autosomal LoF mutations were identified in *SCN2A*, *MED13L*, and *KCNV1. SCN2A* is one of the few genes found recurrently mutated in unrelated patients with ASD and intellectual disability, which is unlikely to occur by chance [10,36,37,62]. *MED13L* was previously associated with intellectual disability and heart defects, and a *de novo* splicing mutation was described in an autistic patient [13,63]. Recently, *de novo* deletions affecting coding regions of *MED13L* were found in two girls presenting a phenotype very similar to the patient we report here, including facial dysmorphism, hypotonia, and development delay, along with ASD [64]. Our findings are consistent with a role of *MED13L* in neurodevelopmental disorders. The third *de novo* and likely pathogenic variant affected *KCNV1*, coding for a potassium channel subunit mainly expressed in the brain and involved in the regulation of two other potassium channels (*KCNB1* and *KCNB2*). Defects in voltage-gated potassium channels have been associated with a variety of neuropsychiatric disorders, including bipolar disorder, schizophrenia, and ASD [12,65-67]. Therefore, our data suggest a role for potassium voltage-gated channels in the etiology of ASD. Finally, we detected a *de novo* missense mutation in the *CUL3*, which is other of the few recurrently *de novo* mutated genes in ASD patients [11,39].

Regarding X-linked mutations, we identified alterations in two genes (*MAOA* and *CDKL5*) previously associated with ASD and intellectual disability. We found a splicing mutation in *MAOA* in a multiplex family with and X-linked pattern of inheritance, with two affected male siblings and a maternal history of psychiatric disease. *MAOA* encodes the protein monoamine oxidase A, which degrades amine neurotransmitters such as dopamine, norepinephrine, and serotonin [68]. Both affected siblings had consistent biochemical alterations of the catecholamine pathway, very mild in their carrier mother, then supporting the pathogenicity of the mutation. A *MAOA* truncating mutation was first described in a Dutch family in 1993, and recently, a second loss of function mutation was found in a family segregating ASD and behavioral problems [69,70]. Our work further strengthens the relation between *MAOA* and ASD. The other X-linked mutation affected a highly conserved amino acid in *CDKL5* and is predicted to be deleterious by different algorithms [71,72]. It was inherited from the healthy mother and also detected in the unaffected sister, who preferentially inactivated the mutated allele. Mutations in *CKDL5* were reported in X-linked infantile spasms syndrome (ISSX) [MIM 308350], atypical Rett syndrome (RTT) [MIM 312750], and Angelman syndrome-like [MIM 105830] [40-42,73,74]. Since the phenotype of our patient is less severe than the one described in males with *CDKL5* mutations, it is possible that the missense variant found in this patient is a hypomorphic allele with a milder effect. The biased X inactivation documented in the sister’s proband could act as a protective factor in females explaining their unaffected status.

While the definition of the potential pathogenicity for ASD mutations could be relatively straightforward in *de novo* and Mendelian cases assuming full penetrance, a greater challenge is the classification of putatively pathogenic heterozygous mutations and rearrangements inherited from unaffected parents. These variants, presumably with incomplete penetrance and variable expression, are thought to contribute to disease risk in an oligogenic model with probable environmental contribution. One of the most common criteria to define potential pathogenicity is the recurrence of mutations in the same gene in unrelated patients. In our small cohort, we detected two additional patients with rare inherited missense mutations in genes with *de novo* LoF mutations in other cases (*SCN2A* and *MED13L*), suggesting that the inherited mutations could also be contributing to the disease. While amorphic alleles might be highly penetrant, hypomorphic missense mutations might have a milder effect just increasing the global burden of risk for ASD. Moreover, the study of common functional consequences of rare variation pointed towards a set of relevant pathways only overrepresented in ASD patients. Among these, there were the PI3K/Akt signaling and the axon guidance which were previously associated to ASD by linkage studies, rare CNVs, genomic mutations, and comorbid ASD conditions [45-48].

Using CNV detection tools on WES data, we identified small rearrangements that were missed by previous molecular karyotype in eight cases, all of them inherited from unaffected progenitors. Some of these rare CNVs could also contribute to the disease in a multiple-hit model, such as the duplications found in *ASMT* as previously proposed [50-52]. Although we did not observe a significant effect on expression in the individual analysis of these rare CNVs, a global analysis showed a tendency for higher expression of genes in duplication type CNVs compared to those in deletions, as expected and described for other ASD-related rearrangements [20,75].

The incorporation of the peripheral blood transcriptomic data provided an important additive value to the identification of molecular biomarkers of ASD, leading to identification of additional mutations and transcriptional consequences. Despite that blood is not the ideal target tissue to study ASD, it is commonly used in neurodevelopmental disorders since it can be easily available for diagnostic testing [76]. In our study, approximately 30% of the rare variants were sufficiently expressed in blood, and 88% of these had a concordant calling in both techniques. By isoform analysis, we found an aberrant transcript in *PTEN*, due to a *de novo* intronic mutation that activates a cryptic splice site. The patient (ASD_36) presents macrocephaly, a feature that is consistently found in ASD patients with mutations in this gene (MIM 605309). Thus, transcriptome sequencing of blood cells was essential to achieve a diagnosis in an additional patient, reaching a final diagnosis yield of 19%. Moreover, the integrative approach also enabled the identification of rare inherited variants with functional consequences that could contribute to the phenotype. As previously suggested, the joint study of genomic and transcriptomic data can be crucial to unravel the mechanism of complex diseases [77,78]. We detected alteration in expression levels in 1.7% of expressed rare variants, inherited in all cases. Overexpression of *MECP2* was found in a patient who had a rare SNP variant in the same gene. Duplication of *MECP2* causes a known duplication syndrome almost exclusively in males with moderate to severe intellectual disability. Overexpression of the gene in peripheral leucocytes was previously described in ASD patients [79] and was also related to aggressive social behavior in schizophrenia [80]. We also identified three rare mutations with concurring overexpression of candidate genes *ANK3* [81,82], *CREBBP* [83,84], and *SEMA6B* [85] in a single patient. They were inherited from both progenitors and they could contribute to ASD in an additive manner. Additionally, we also detected monoallelic expression of the wild-type allele associated with ten rare inherited truncating mutations suggesting non-sense mediated decay (NMD) in which the functional haploinsufficiency could contribute to the phenotype such as the alteration in *ALG9* [MIM 606941] and *RIT1* [MIM 609591] previously involved in neuropshychiatric conditions [86-88]. Finally, allele-specific expression analysis revealed the alteration of 68 genes (or specific transcript variants) with monoallelic expression but no *cis*-element responsible for it. This phenomenon was found to be more common for autosomal and X-linked genes in ASD patients than in controls in the brain and other tissues [89,90]. Allele-specific expression can be caused by unidentified *cis*-acting elements, including genetic or genomic mutations in the promoter or regulatory regions and epigenetic marks. Thus, some of identified genes with allele-specific expression might contribute to ASD. In fact, two of them have been previously associated (*MTOR*) [91] and/or are great candidates (*FUS* and *TAF1C*) [92,93]. Ongoing efforts to define the extent of expression variation in large numbers of healthy controls such as Geuvadis and GTEx will help to better clarify the deregulated genes found in individual patients that are related to disease.

## Conclusions

In summary, our data reinforces the clinical utility of NGS in establishing the cause of ASD. Although transcriptome sequencing is limited by the genes expressed in the analyzed tissue, it has proven to be very useful in combination with WES. Their integration has determined the molecular defect compatible with highly penetrant monogenic ASD forms in 19% of the studied cohort. Blood transcriptomic data also revealed functional correlations of genetic variants, including changes in splicing, expression levels, and allelic expression, as well as novel candidates with allelic imbalance. Considering the likely multifactorial etiology of most cases of ASD, data interpretation of the potentially deleterious variation inherited from unaffected progenitors still represents a challenge. Joint analysis of transcriptome sequencing and deregulated pathways by rare genetic variation can help to elucidate the genetic contribution of this complex disorder and define deregulated mechanisms. The identification of all genetic contributors in each patient will help to establish genotype-phenotype correlations, define specific disorders, and identify pathophysiologic mechanisms that can guide pharmacological approaches.

## Additional files

Additional file 1: Figure S1. Distribution of WES variants per patient after filtering. Grey bars correspond to missense mutations, whereas red bars correspond to LoF mutations. **Figure S2.** A) Pedigree of the family ASD_16 compatible with X-linkage inheritance. B) Validation by Sanger sequencing of the hemizygous *MAOA* splicing mutation (c.1438-2A > G) in the proband and brother as well as the heterozygous mother. C) Schematic representation of the aberrant *MAOA* transcript detected in blood mRNA of the proband, generated by the use of a cryptic acceptor splice site in intron 15 of the gene. **Figure S3.** Distribution of z-score values among genes contained in all Copy Number Variant regions (CNVs) detected by XHMM in exome data. **Figure S4.** Boxplot showing distribution of RNA AB ratios for all informative SNPs located in genes showing ASE in ASD patients. Additional file 2: Table S1. List of ASD candidate genes. **Table S2.** Variants selected for validation and segregation after filtering. **Table S3.** X-linked variants selected for validation and segregation studies. **Table S4.** Cathecolamine and metabolites in 24 hours urine. **Table S5.** Recurrently mutated genes with no LoF reported in EVS. **Table S6.** Rare CNVs selected for validation and segregation. **Table S7.** Rare mutations with changes in expression levels. **Table S8.** Detected nonsense mediated decay events. **Table S9.** Genes with allele specific expression.

## Abbreviations

ADHD: attention deficit hyperactivity disorder
ADI-R: Autism Diagnostic Interview Revised
ASD: autism spectrum disorders
ASE: allele-specific expression
CNV: copy number variants
CPDB: ConsensusPathDB
DGV: Database of Genomic Variants
DP: depth of coverage
DSM-IV: Diagnostic and Statistical Manual of Mental Disorders
EVS: Exome Variant Server
FPKM: fragments per kilobase of transcript per million mapped reads
GATK: Genome Analysis Toolkit
IGV: Integrative Genome Viewer
ISSX: X-linked infantile spasm syndrome
LoF: loss of function
MAF: minimum allele frequencing
MLPA: multiplex ligation probe amplification
NGS: next-generation sequencing
PAR1: pseudoautosomal region 1
PBMCs: peripheral blood mononuclear cells
PDD-NOS: pervasive developmental disorder not otherwise specified
RTT: Rett syndrome
SNV: single nucleotide variant
WES: whole-exome sequencing
XCI: X chromosome inactivation
