# Supplementary material for: Integrated analysis of whole-exome sequencing and transcriptome profiling in males with autism spectrum disorders
Source: Mol Autism. 2015 Apr 15;6:21. doi: 10.1186/s13229-015-0017-0 (PMC4427998; doi:10.1186/s13229-015-0017-0)

Figure S1. Distribution of WES variants per patient after filtering. Grey bars correspond to missense mutations, whereas red bars correspond to LoF mutations.

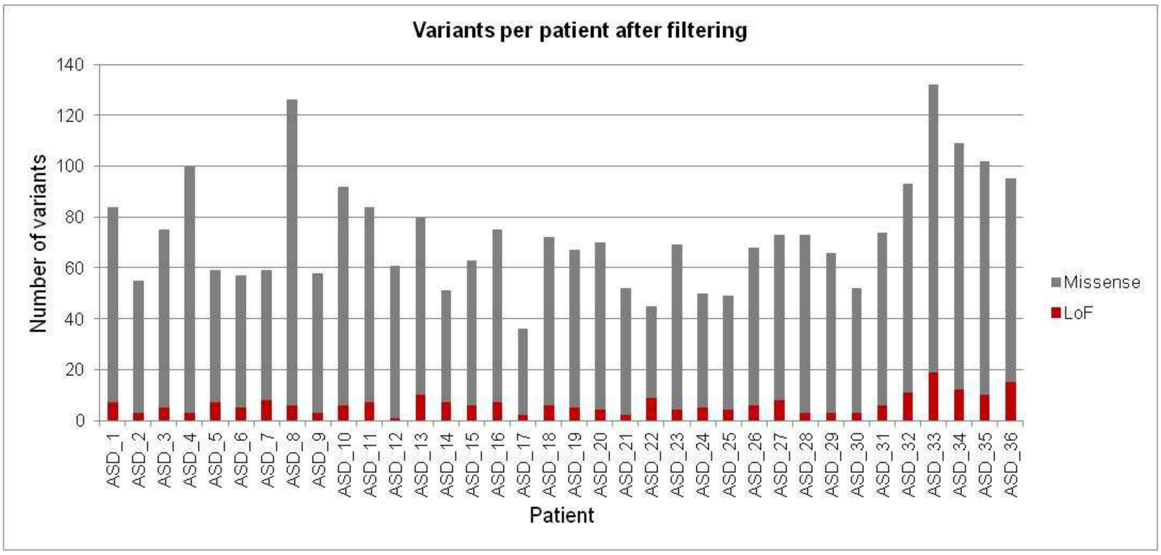

Figure S2. A) Pedigree of the family ASD_16 compatible with X-linkage inheritance. B) Validation by Sanger sequencing of the hemizygous *MAOA* splicing mutation (c.1438-2A>G) in the proband and brother as well as the heterozygous mother. C) Schematic representation of the aberrant *MAOA* transcript detected in blood mRNA of the proband, generated by the use of a cryptic acceptor splice site in intron 15 of the gene.

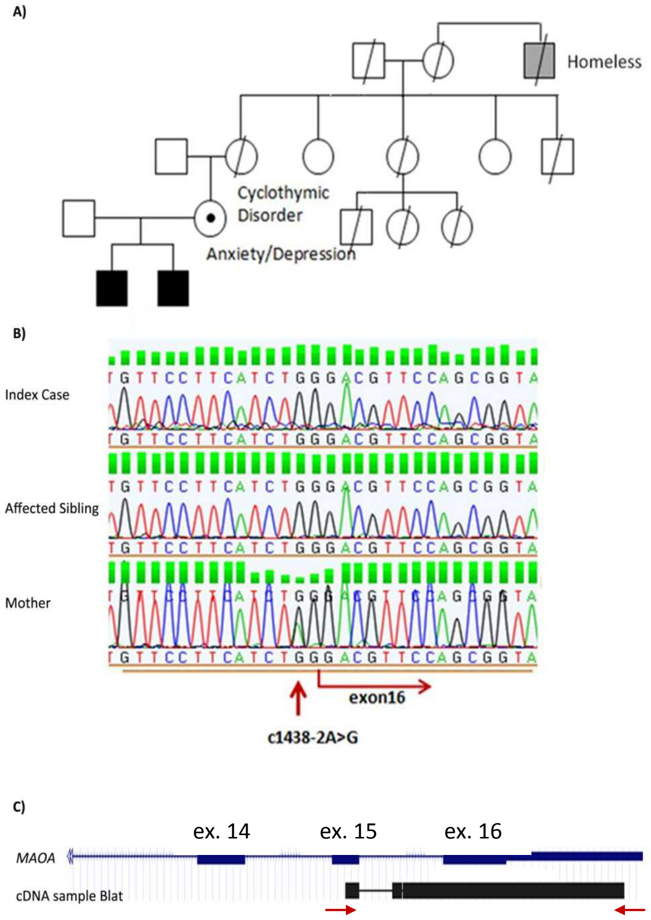

Figure S3. Distribution of z-score values among genes contained in all Copy Number Variant regions (CNVs) detected by XHMM in exome data.

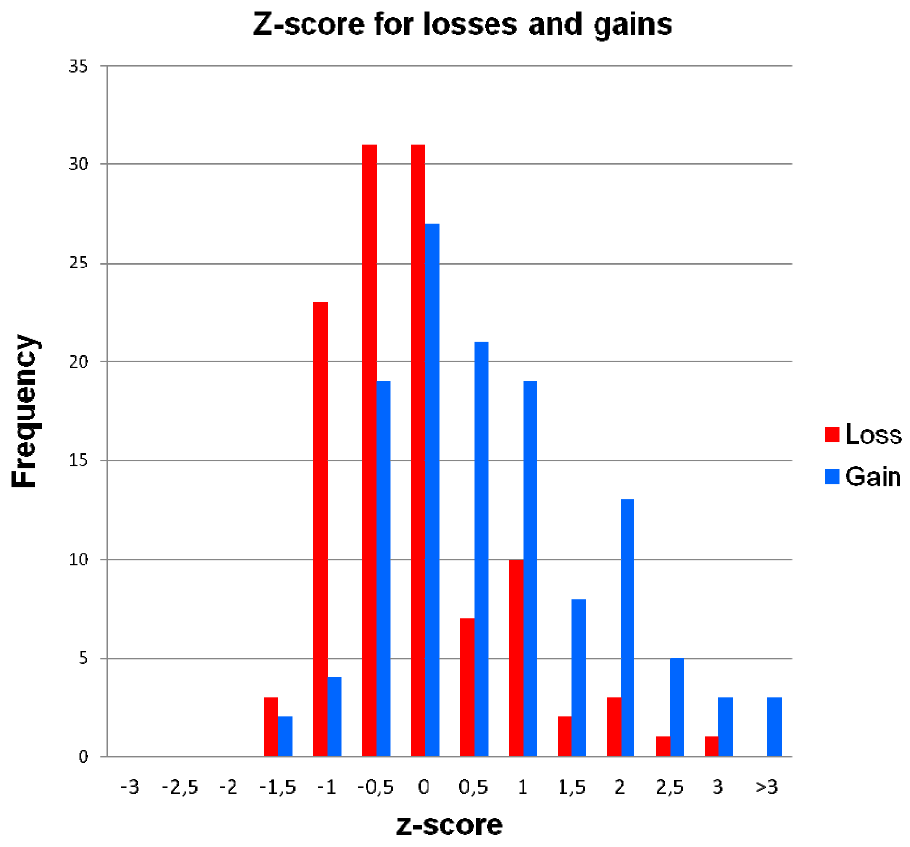

Figure S4. Boxplot showing distribution of RNA AB ratios for all informative SNPs located in genes showing ASE in ASD patients.

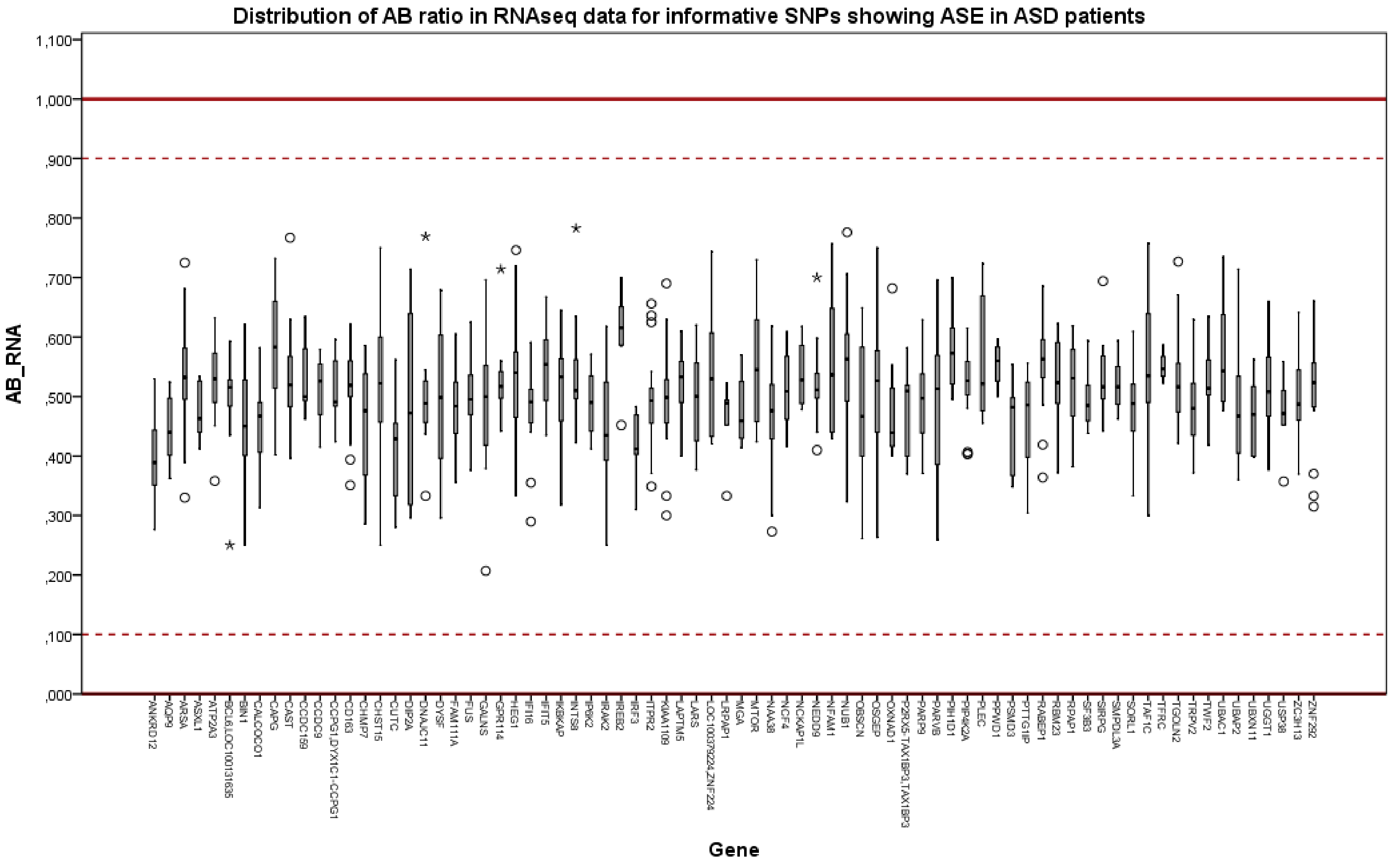

Supplement: Additional file 1: Figure S1. — Distribution of WES variants per patient after filtering. Grey bars correspond to missense mutations, whereas red bars correspond to LoF mutations. Figure S2. A) Pedigree of the family ASD_16 compatible with X-linkage inheritance. B) Validation by Sanger sequencing of the hemizygous MAOA splicing mutation (c.1438-2A > G) in the proband and brother as well as the heterozygous mother. C) Schematic representation of the aberrant MAOA transcript detected in blood mRNA of the proband, generated by the use of a cryptic acceptor splice site in intron 15 of the gene. Figure S3. Distribution of z-score values among genes contained in all Copy Number Variant regions (CNVs) detected by XHMM in exome data. Figure S4. Boxplot showing distribution of RNA AB ratios for all informative SNPs located in genes showing ASE in ASD patients. [file 13229_2015_17_MOESM1_ESM.pdf]
